# Interosseous Malignant Melanotic Nerve Sheath Tumor of the Sacrum Treated With an Innovative Reconstruction Technique

**DOI:** 10.7759/cureus.64820

**Published:** 2024-07-18

**Authors:** Fahad M Okal, Badr E Hafiz, Ali Alassiri, Zeyad Alamri, Wael Alshaya

**Affiliations:** 1 Neurosurgery Division, Department of Surgery, King Abdulaziz Medical City, Ministry of National Guard - Health Affairs, Jeddah, SAU; 2 Department of Neurosurgery, King Faisal Specialist Hospital and Research Centre, Jeddah, SAU; 3 Neuropathology Division, Department of Pathology, King Abdulaziz Medical City, Ministry of National Guard - Health Affairs, Riyadh, SAU; 4 Pediatric Neurosurgery Division, Department of Pediatric Surgery, King Abdulaziz Medical City, Ministry of National Guard - Health Affairs, Riyadh, SAU

**Keywords:** case report, melanotic, extramedullary, malignant, tumor, nerve sheath

## Abstract

Intraosseous malignant melanotic nerve sheath tumors are extremely uncommon peripheral nerve sheath tumors that typically present with benign clinical and histopathological features but with more aggressive long-term behavior. These tumors commonly originate from the dorsal nerve roots, sympathetic chain, cranial nerves, and lumbar plexus but may be found throughout the body. It usually presents with gradual compressive symptoms over months to years, like the typical presentation of schwannomas. The mainstay of treatment is surgical resection, with gross total resection recommended. Additional treatment with adjuvant therapy for recidivist or metastatic disease is less well-defined due to the rarity of these tumors. Adjuvant radiation following resection that is not gross total or that accomplishes clear surgical margins is advocated by some authors, although there is no strong evidence for it in the literature.

In this report, we describe an extremely rare case of gradual onset, progressive spinal cord dysfunction in a patient with a lumbosacral intraspinal malignant melanotic nerve sheath tumor with bony invasion that was treated with an innovative reconstruction technique that to the best of our knowledge is first described in our paper. The patient achieved excellent functional and neurological outcomes after the surgical excision and reconstruction.

## Introduction

Malignant melanotic nerve sheath tumors (MMNST) originate from neuroectoderm and have characteristic Schwann cells with melanin-pigmented cytoplasm [1]. Melanin pigment in the pia mater can be seen as it occurs in some tumors, such as melanomatous meningiomas [1]. MMSNT is an extremely rare primitive nerve sheath tumor, as it accounts for 0.5% of all nerve sheath tumors [2]. In 2013, the World Health Organization classified melanotic schwannoma as a benign tumor, but in 2020 for the classification of soft tissue tumors and in 2021 for the classification of central nervous system tumors, the term “melanotic schwannoma” was revised to “malignant melanotic nerve sheath tumor (MMNST)” due to its malignant behavior [2]. MMNST can be divided into psammomatous (affecting spinal nerves and paraspinal ganglia) and non-psammomatous (affecting autonomic nerves of the viscera and cranial nerves) types [2]. However, they rarely extend from the spinal nerve roots to the spinal epidural space [2]. Spinal MMNST occurs in the cervical (22.2%), thoracic (30.5%), and lumbosacral (47.2%) regions [2,3]. The intramedullary type is rarely seen [2]. Malignant and metastatic potential are displayed by MMNST depending on the histology [3]. Symptoms and signs are based on the site of the tumor. Classically, it arises from the nerve sheath, which explains the paresthesia, weakness, and pain upon presentation [3]. MMNST can be misdiagnosed with degenerative disc diseases due to its presentation with non-specific back pain combined with radicular pain [4]. Further growth of the tumor leads to the worsening and progression of motor and sensory signs and symptoms [3]. Magnetic resonance imaging is an important diagnostic tool that can aid the diagnosis [4]. MMNST should be included in the differential diagnosis for any patient who presents with back pain that is associated with slowly progressing motor and sensory signs and symptoms [4].

## Case presentation

**Figure 1:**
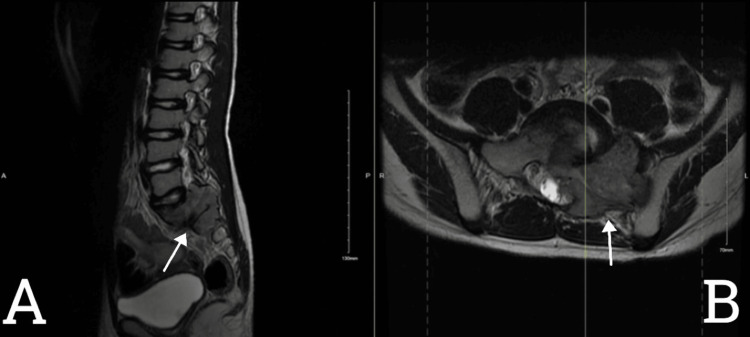
The MRI lumbosacral T2WI sagittal view (A) and axial view (B) An extensive L5–S2 lesion causing destruction of the left side of sacral ala and posterior elements of S1–S2 can be seen in both sagittal and axial views. It extends to the central canal, S1–S2 neural foreman, and left sacroiliac articulation. There is mild left paravertebral muscular extension and atrophy of the left pisiform muscle, likely related to chronic nerve compression by the mass. There are also signal changes manifested by T2WI hyperintensities and enhancement, for tumor involvement.

The patient is a seven-year-old boy not known to have any previous medical illnesses. His parents sought medical advice two years ago at a private hospital with a complaint of on/off low back pain with no mechanical association. No established diagnosis was made then. His pain became more constant and severe, making attending school difficult because of his significant discomfort in sitting positions with left-sided sciatica lasted for seven months. There was no associated lower-limb weakness, urinary incontinence, or sensory complaints. At the time of examination, he was a healthy-looking boy with a normal gait, no obvious deformity, and intact motor power. He underwent a CT-guided biopsy in another facility which was suggestive of a melanotic nerve sheath tumor. An MRI was performed (Figure 1), revealing an extensive lumbosacral lesion occupying the disc space of L5-S2 with bony erosion.

**Figure 2:**
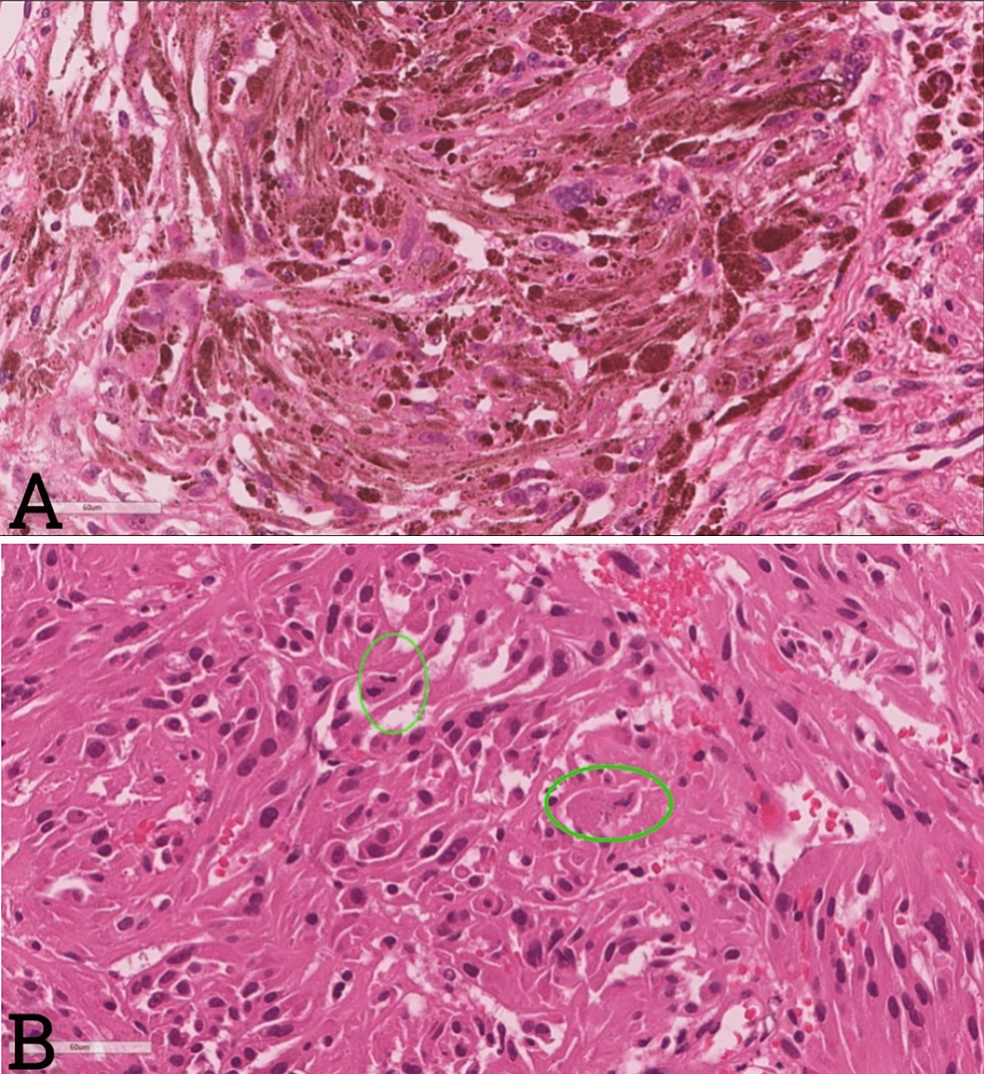
The hematoxylin-and-eosin-stained sections (A) A high-power view shows the heavy melanin deposition and visible nucleoli. (B) Mitotic figures (green circles) are observed with ease in the less pigmented areas.

**Figure 3:**
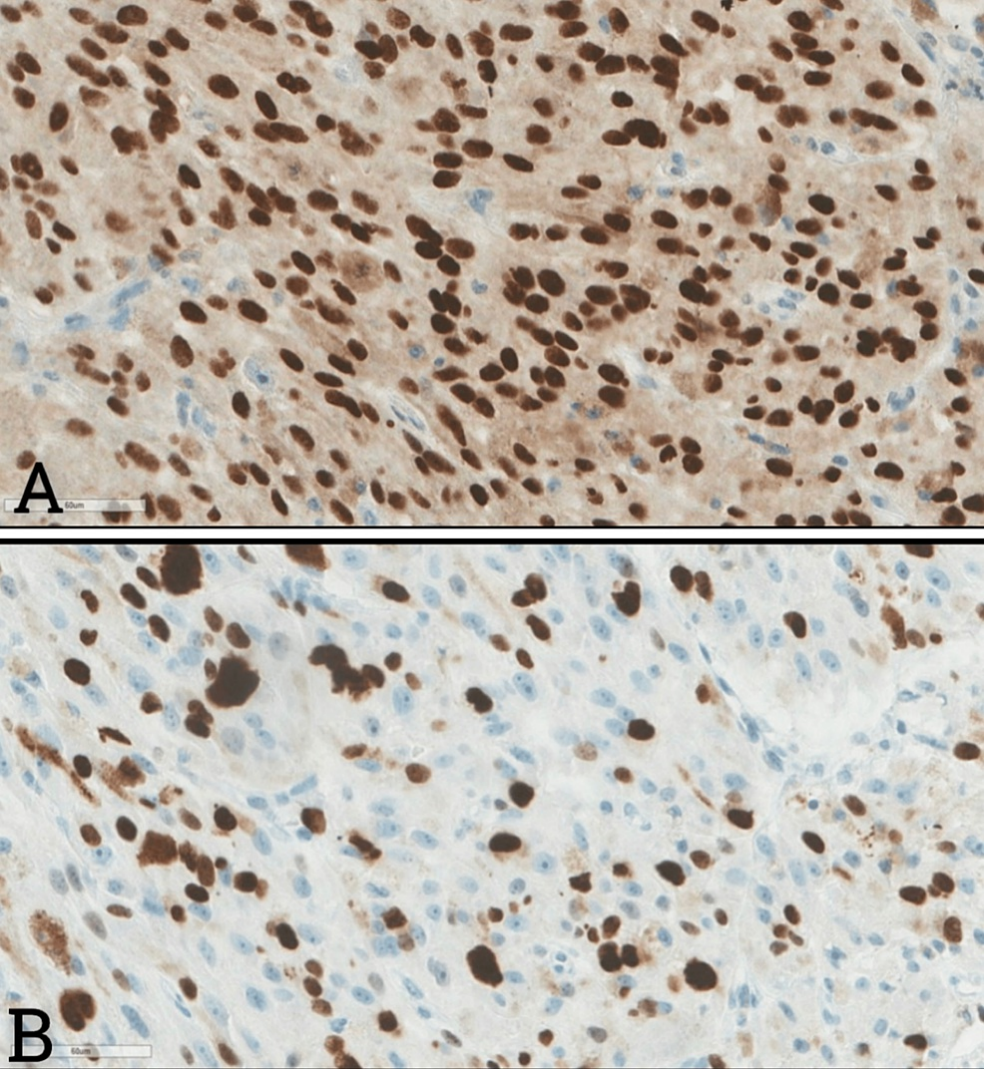
The Immunohistochemistry sections A) Tumor nuclei immunoreacted for SOX-10 with strong intensity. B) Up to a 40% proliferation rate was noticed in most zones labeled Ki-67 (clone MIB-1).

A computed tomography scan of the chest, abdomen, and pelvis and a whole spine MRI ruled out other primary sources of the lesion. The patient was planned for surgical resection of the lesion and fixation with reconstruction. Left L5 laminectomy and gross total resection (GTR) of the tumor with instrumentation were performed by placing L4-S1 pedicle screws and iliac crest screws, with an S1 cage applied horizontally and attached to the left iliac crest with bone dust for better fusion. Intraoperatively, the lesion was darkly pigmented, resembling melanoma, and extensively bloody, and massive transfusion protocol was activated. The lesion had some dural invasion with intradural extension. The specimen was sent for pathological evaluation to aid in establishing the diagnosis (Figures 2-3). 

The hematoxylin-and-eosin-stained sections prepared from both the preoperative core biopsy and the subsequent debulking specimen revealed a heavily melanin-pigmented spindled and epithelioid neoplasm (Figure 2A). Some of the tumor nuclei exhibited prominent macronucleoli, and mitotic figures were identified with no difficulty in the less pigmented areas (Figure 2B). By immunohistochemistry, the tumor cells were immunoreactive for SOX-10 (Figure 3A). S100, HMB-45, and Melan-A had a roughly 40% Ki-67 labeling index (Figure 3B). As such, the differential diagnosis lay between an MMNST versus a malignant melanoma. To sort out this differential with certainty, the tumoral tissue was subjected to comprehensive genomic profiling (Foundation®One Heme, Foundation Medicine Inc., Cambridge, MA), which revealed PRKAR1A loss that is diagnostic of a malignant melanotic nerve sheath tumor. 

**Figure 4:**
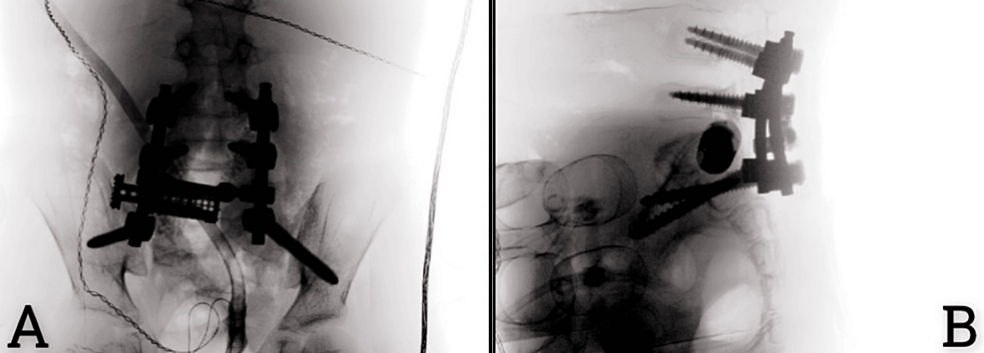
(A) Anteroposterior and (B) lateral lumbosacral X-rays show L4/5 instrumentation with an S1 expandable cage horizontally placed from the S1 body to the sacro-illiac joint on the left side, with bilateral S2 iliac screws containing a horizontally placed cage in S1

The surgery was uneventful and without any intraoperative or postoperative complications. The patient was shifted intubated to the pediatric intensive care unit and extubated on the next day postoperatively without any neurological deficits. A postoperative lumbosacral X-ray was performed (Figure 4). 

As shown in the X-ray of the lumbosacral spine, a new surgical technique with a cage was implemented to stabilize the lumbosacral area with preserved alignment along the iliac screw’s implementation. The patient tolerated the procedure well and was discharged on the fourth postoperative day in a lumbar brace. 

**Figure 5:**
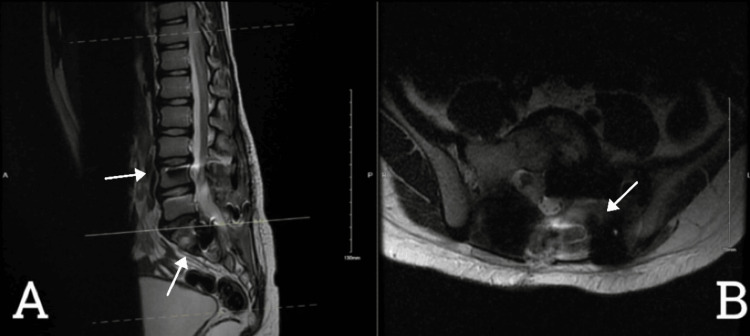
The MRI lumbosacral T2WI sagittal view (A) and axial view (B) The artifact along the area of concern secondary to the screws and rod fixation can be seen in the above panels. Again, evidence of postsurgical changes and the area of the bone graft anterior to the left-most inferior screw can be seen. At a similar level along the lateral sacral crest of S2, there is an area of high T2 and low T1 signal intensity with post-contrast showing mainly thick peripheral enhancement with a central non-enhancing component. The lesion appears to invade the S2 neural foramina. It measures approximately 1.5 × 2.1 cm (AP and transverse dimensions respectively). The remainder of the visualized osseous structures are unremarkable. The visualized spinal cord shows normal morphology and signal intensity.

The patient was seen in the clinic after two months. He was doing well, his pain had dramatically improved, he was able to walk independently and he was neurologically intact. A follow-up postoperative MRI was performed (Figure 5) showing the artifact in the site of the screws and rod fixation, normal postoperative changes, and the area of the bone graft anterior to the left-most inferior screw, as well as excellent alignment. 

**Figure 6:**
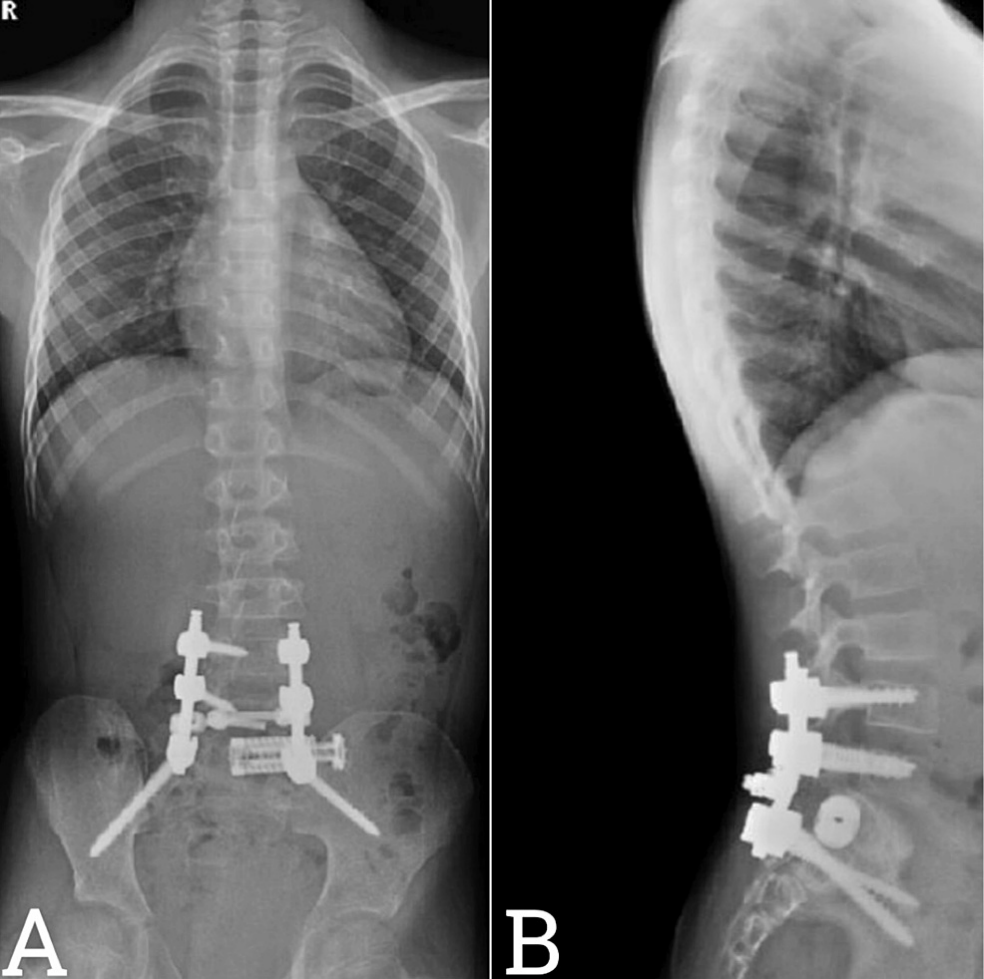
(A) Anteroposterior and (B) lateral whole spine X-rays show L4/5 instrumentation with bilateral S2 iliac screws with a horizontally placed expandable cage at the left L5 hemi corpectomy site to the left iliac crest

The patient was referred to the medical neuro-oncology team to start the neoadjuvant treatment plan. He received local radiation 31 fraction as planned by the neuro-oncology team. At his one-year follow-up, the patient was free of pain, able to walk independently, and had no new symptoms or evidence of tumor recurrence in his MRI and positron emission tomography scan. He underwent another whole spine X-ray (Figure 6). 

As shown in the X-ray of the whole spine, the implemented cage is in place with preserved alignment along the iliac screw’s implementation as it was in the immediate baseline postoperative lumbosacral X-rays. His CT lumbosacral spine showed complete fusion after the first postoperative year. He will have continuous follow-up appointments with serial imaging in our clinic to follow his clinical condition. 

## Discussion

A slowly growing painless mass or lump could be the presentation of melanotic nerve sheath tumors [5]. The symptoms are varied and include sensory alterations, numbness, paresthesia, and weakness [6]. The diagnosis can be made by imaging studies in combination with histopathological studies [6]. Carney syndrome can be the cause of MMNST in the young population [6]. Sympathetic ganglia and spinal nerves are the common origin of MMNST as a single lesion [7]. Other sources reported in the literature are the cranial nerves, sympathetic chain, cerebellum, peripheral nerves, choroids, orbit, soft tissues, heart, esophageal wall, pancreas, parotid gland, alveolar nerves bones, and trachea [8]. These tumors can show aggressive behavior as they invade the surrounding structure and metastasize to other organs of the body [8]. Contrary to common schwannomas, MMNSTs are more aggressive in terms of local invasion and recurrence, as well as the potential metastatic behavior that takes place primarily in the pleura and lungs but also in the endocardium, pericardium, diaphragm, mediastinum spleen, liver and bones [8]. Melanotic nerve sheath tumor treatment options are surgery and/or neoadjuvant chemo-radiotherapy or watchful waiting [7,9]. Surgery is the cornerstone of treatment for MMNST, with GTR being the gold standard for all subtypes [9]. During surgery, maximal safe resection is necessary to prevent spinal cord injury, which will worsen the outcomes of the surgery, although the extent of resection is primarily associated with the survival and recurrence rate and the necessity of neoadjuvant therapy [6,9]. In our presented case, the spine was destabilized by tumor resection and required instrumentation from L4/5 and bilateral iliac screws with a horizontally placed expandable cage at the left L5 hemi corpectomy site to the left iliac crest with bone dust for better fusion. To our knowledge, this new innovative technique of placing the cage horizontally is first described and published in our paper.

Due to the rarity of melanotic nerve sheath tumors, prognoses and long-term outcomes can vary. Regular follow-ups and monitoring are necessary to detect any potential recurrence or metastasis, as MMNSTs have a higher local recurrence rate than other schwannomas.

## Conclusions

MMNST should be considered in the differential diagnosis for all age groups, especially when melanin is seen in an intradural spinal tumor. Intradural extramedullary lesions in the region of the lumbosacral spine can include myxopapillary ependymoma, paraganglioma, nerve sheath tumor, meningioma, and metastasis. The MMNST diagnosis is based on clinical, histopathological, and instrumental findings. The histological diagnosis requires careful differential considerations between neurofibroma, pigmented dermatofibrosarcoma protuberans, melanocytoma, and malignant melanoma. Surgical resection is the mainstay and cornerstone of management. Different operative strategies can be employed for reconstruction and stabilization, in addition to instrumentation that can be applied, as in the technique described in our surgical approach to manage this aggressive tumor. Considering the high recurrence rate, adjuvant radiation therapy should be taken into consideration, especially when GTR is unachievable.
